# Is It Worth Publishing in a Medical Students’ Journal? Insights From a 10-Year Journey

**DOI:** 10.5195/ijms.2021.1309

**Published:** 2021-12-16

**Authors:** Francisco J. Bonilla-Escobar, Ciara Egan, Mihnea-Alexandru Găman

**Affiliations:** 1 MD, MSc, PhD(c). University of Pittsburgh, Instituto for Clinical Research Education, Department of Ophthalmology, Pittsburgh, PA, United States. Science to Serve the Community, SCISCO Foundation / Fundación SCISCO, Colombia. Service of Ophthalmology, Universidad del Valle, Cali, Colombia. Editor in Chief, International Journal of Medical Students, IJMS; 2 Medical Student, Humanitas University, Humanitas Research Hospital, Milan, Italy. Deputy Editor, IJMS; 3 MD, PhD student. “Carol Davila” University of Medicine and Pharmacy, Bucharest, Romania. Department of Hematology, Center of Hematology and Bone Marrow Transplantation, Fundeni Clinical Institute, Bucharest, Romania. Scientific Editor, IJMS

Very little has been said about young scientists publishing in Journals that are led and carried forward by medical students or recently graduated physicians themselves. What has been said, however, is not always supportive. For some, these Journals should not exist, arguing that medical student research is for the sole purpose of training or rather, it should be aimed at reaching the level of rigor and impact held by other professional journals. For others, the lack of indexing and impact make these Journals a place where potential visibility and recognition is lost. Yet, there is a community of believers who see us as a way to improve science by training the next generation from the onset of their careers. A for-students, by-students Journal is the crossroads between publication, and therefore visibility, of student research, and a training ground for the ins and outs of peer-review and the writing standards held in science and medicine. With time, the rigor and excellence of these journals grows, and indexing follows.

The future generation of physician scientists is in dire need of growth, as there are more of them retiring that there are newly graduating.^1^ Medical student research is therefore a need of the whole world, and it should begin in the early stages of their careers. Yet, research curriculums in medical schools have a wide spectrum of aims, teaching techniques, and resources. This leads to vast differences in the opportunities available to students depending on where they study. The world of research and investigation fantastically breaks down these walls and engaged, motivated students learn to find their way. Student journals can be the places where these boundaries are taken down and allow students from any background and institution to mature and succeed.

At the IJMS we strive to be a place for all medical students to come and join a community of student researchers aiming at excellent, publishable manuscripts. Working with medical students’ research from all over the world is inspiring.^2^ Every day we receive research and experiences that connect us as a community and push the Journal towards higher levels of commitment and quality of work. The community of authors that we have seen growing steadily in the last couple of years has its repercussions in both the number of articles and issues we publish. For this ninth volume, we have increased the number of issues to four per year to better serve our community. In addition, we just went through a new expansion of our Associate and Student Editors in order to keep up with the submissions. This growth is moving use forward in our plans to be indexed. We are not yet indexed in the National Library of Medicine of the United States, but it is on the horizon for us.

Being a student or recent graduate and working to keep a journal for students alive is not an easy task. There is not a class in medical school about how to be an editor or a peer-reviewer. Classically, these skills are learned on the go and through trial and error. The Journal day-to-day work, its online platform for publication, and its standards and scope truly are realized and improved every day. Our peer-review process has been normalized across the board through online courses that train students on how to review articles systematically and thoroughly. On a larger scale, the Journal is guided by organizations we apply to join (i.e., COPE) and by the guidelines we must adhere to for our publications (i.e., University of Pittsburgh Library System). At IJMS, we aim for everyone involved with the Journal, editors and authors alike, to grow and mature as young scientists with each publication and issue we have.

As a young student-scientist, is it worth your time and effort to publish in a Journal for medical students? We believe so. Beyond visibility of your work in the online research world, you join a community that wants you to succeed as a researcher. You will be supporting your fellow medical students by sustaining this community. Furthermore, with time and a commitment to excellence, the IJMS will grow in its impact and indexing. We are a place of dialogue and exchange of ideas for students, and we strive for medical students’ and recently graduated physicians’ research work to always have a place in the medical scientist arena.

A step away from our 10^th^ anniversary, publishing non-stop, we celebrate this with the announcement that our aim to be indexed in PubMed/MEDLINE is stronger than ever and that the Journal is thriving in its publishing indicators. Only in the last 12 months (January 1^st^ 2021 up to December 6^th^ 2021), we have had 317 submissions, an acceptance rate of 19%, 123 days to acceptance since submission, and 29 days from submission to rejection. If we compare these numbers with the previous year, we had 316 submissions, an acceptance rate of 33%, 180 days from submission to acceptance, and 110 days to rejection. In addition, our next issue (Volume 10 Issue 1 of 2022) is almost ready for publication more than three months before the publication deadline.

The above-mentioned goals and achievements could not have been reached without our team of Associate Editors, Student Editors, Layout Editors, Communications and Public Relationships Committee, and Editorial Board. In this Editorial, we acknowledge the members of our Editorial Team who participated the most, were permanently active in the Journal, and provided exceptional feedback or performed outstandingly in their assigned tasks: ***Sohaib Hasseb*** as the Associate Editor of the Year 2021, ***Adnan Mujanovic**, and **Joseph Tonge*** as the Student Editors of the Year 2021, ***Sajjad Ali*** as the Layout Editor of the Year 2021. A special acknowledgement goes to ***Madeleine J. Cox and Andrew Thomas*** who besides being excellent Student Editors are being promoted to Associate Editors. We make a special mention to ***Dr. Georgiana Farrugia*** as our outgoing Director of Communications and Public Relationship. She has done an extraordinary job in this position and our social networks status are evidence of her legacy. The International Journal of Medical Students is probably one of the few journals that requires the entire Editorial Team to be certified in the art of peer-review. Thus, all our staff has graduated from The Publons Academy, a peer-review course within the Clarivate Analytics (former Institute for Scientific Information, ISI), and has received a certified status of peer-reviewer after having evaluated two papers under the supervision of a mentor.^3^ Currently, as the Publons Academy was recently integrated into the Web of Science Academy, we request our editorial team members to complete the following courses: *An introduction to Peer-Review, Reviewing in the Sciences, Good Citation Behavior*, and *Co-reviewing with a Mentor*.^4^ The effort invested into the evaluation of manuscripts submitted to the IJMS certifies that we are dedicated to publishing only work of the highest quality.

This issue is full of relevant research for medical students and the entire scientific community, including research on COVID-19 and other relevant topics. This last issue of 2021 revolves around seven original articles, one short communication, one case report and four experiences written by medical students from all over the world.

Apart from this editorial, we reinforce our support for climate change, as discussed at the United Nations Climate Change Conference (COP26) held recently in Glasgow. Thus, we have decided to publish an editorial piece on climate change and biodiversity that begins with an interesting motto: “The Health Community Must Step Up Its Efforts to Hold Countries Accountable for Reducing Greenhouse Emissions and Promoting Adaptation”.^5^

The COVID-19 pandemic remains the focus of five original research articles published in this issue, with a peculiar emphasis on the importance of mental and psychological health during these difficult times. Babatunde et al. evaluated the impact of the COVID-19 lockdown on depression severity and the use of drugs among university students from Ibadan, Nigeria, reporting that symptoms of mild depression, measured using the Patient Health Questionnaire (PHQ-9), were experienced by approximately 41% of the subjects who partook in the study. Fortunately, the use of psychoactive substances was low and was not influenced by the lockdowns enforced during the pandemic.^6^ Depression, anxiety, and psychological stress were fairly common in another investigation recruiting adults from India (24%, 14%, and 17%, suffered from the aforementioned ailments, respectively) as evidenced by Prakash et al.^7^ Jenkins and Grasso also presented interesting data regarding the frequency (nearly 38%) of serious adverse pandemic-related experiences (e.g., increased conflict, less physical activity, frequent substance use) in a sample of medical students from the United States of America. Students who spent more time caring for patients diagnosed with the SARS-COV-2 infection were more likely to deal with such psychological distress and to suffer from anxiety, depression, and post-traumatic stress disorder.^8^

Odeyemi et al. explored the knowledge, attitude, and practices towards the preventive strategies against the COVID-19 pandemic among young adults from Nigeria. The researchers revealed that there is optimism that the pandemic will eventually be controlled; however, the use of face masks by the respondents was influenced by their level of education, ethnicity, and other factors.^9^ Ethnicity was also related to the risk of acquiring COVID-19 in a sample of nearly 2,000 adults from the United States. Hispanics were more likely to test positive for SARSCOV-2 and this relationship was mediated by social determinants of health risk factors, as reported by Verdini et al.^10^ Furthermore, due to the importance of involving any available healthcare worker in the fight against the COVID-19 pandemic, Bernard et al. proposed a pandemic leadership model for medical students based on several overarching themes, namely Communication, Other-Orientation, Personal Characteristics, Decisive Action, and Use of Information.^11^

The second half of the issue is more focused on medical students and education. Zeller et al. prospectively compared pocket-sized ultrasound and cardiac auscultation in diagnosing cardiac valve pathologies by medical students, revealing that training in the former technique improves their ability to identify heart valve disorders.^12^ Silver et al. implemented a series of storytelling events on the topic of formative medical experiences in order to combat burnout and promote mindfulness and wellness which benefited all participants, but in particular medical students.^13^ In their case presentation, Mohamed et al. pointed out that the use of ring pessaries, a conservative approach method employed in the treatment of pelvic organ prolapse, may results in a severe but rare complication, i.e., rectovaginal fistula.^14^ The four experience pieces of the issue span a variety of relevant topics in the career of any healthcare professional: gender bias, gender equity and the prevalence of stereotypes in clinical medicine and surgery, volunteering in times of war, and health promotion using medical education.^15–18^

We move into the New Year with high expectations and hope to keep this Journal flourishing and growing. We wish everyone in the community Happy Holidays. We resolve to continue growing the Journal, and we look forward to you joining us in this adventure. Cheers!
